# Effect of *Salmonella enteric* Serovar Typhimurium in Pregnant Mice: A Biochemical and Histopathological Study

**DOI:** 10.4021/gr441w

**Published:** 2012-05-20

**Authors:** Geeta Shukla, Ishita Verma, Lalita Sharma

**Affiliations:** a Department of Microbiology, Panjab University, Chandigarh- 160014, India

**Keywords:** *Salmonella*, Pregnancy, Lipid peroxidation, Liver enzymes, Aspartate aminotransferase, Alanine aminotransferase, Alkaline phosphatase, Histopathological alterations

## Abstract

**Background:**

Food borne infections caused by *Salmonella enterica* species are increasing globally and pregnancy poses a significant threat in developing countries, where sanitation facilities are inadequate. Thus, the present study was designed to delineate the effect of *Salmonella* infection during pregnancy.

**Method:**

Pregnant, BALB/c mice were challenged orally with *Salmonella enterica* serovar Typhimurium on gestational day 10 and were monitored for bacterial load, hepatic injury, histopathological alterations vis-a-vis oxidant and antioxidant levels.

**Results:**

Pregnant-*Salmonella*-infected mice had higher bacterial translocation in the liver, spleen as well as liver enzymes mainly aspartate aminotransferase, alanine aminotransferase and alkaline phosphatase compared with *Salmonella-*infected mice. The levels of lipid peroxidation were significantly higher in all the organs of both pregnant-*Salmonella-*infected and *Salmonella*-infected mice compared with control mice. However, the activities of antioxidant enzymes (reduced glutathione, superoxide dismutase and catalase) were lower in the liver, spleen and placenta of pregnant, pregnant-*Salmonella*-infected and *Salmonella-*infected mice compared with control mice, but the decrease was more in pregnant-*Salmonella*-infected mice indicating depression of antioxidant defense system. Histopathologically, pregnant-*Salmonella*-infected mice had more architectural damage in the liver, spleen and placenta compared with other groups.

**Conclusion:**

Pregnancy makes the host more vulnerable to typhoid fever by affecting the physiology of pivotal organs and highlighting the importance of early and prompts diagnosis so as to avoid the further materno-fetal complications.

## Introduction

Pregnancy is the period of intense biological transformation as almost from the moment of conception, it is the mother and her newborn, which are susceptible to various kinds of diseases. Pregnancy does have deleterious effect on the outcome of infections such as leishmaniasis, malaria, toxoplasmosis, listeriosis and typhoid [1, 2]. Moreover, bacterial enteric diseases are one of the major public health problems throughout the world and among those caused by *Salmonella enteric* serovar Typhi are most frequent and serious in developing nations with poor hygienic conditions, whereas nontyphoidal intestinal disease caused by *Salmonella enteric* serovar Thyphimurium a food borne pathogen, is of global concern [3]. *Salmonella* species infects a variety of hosts and causes broad spectrum of diseases, ranging from acute self-limiting diarrhoea to bacteraemia and enteric fevers [4, 5].

Typhoid fever is endemic in Asia, Africa, Latin America, the Caribbean, and Oceania and about 80% of cases accounts from Bangladesh, China, India, Indonesia, Laos, Nepal, Pakistan, or Vietnam infecting roughly 21.6 million people (incidence of 3.6 per 1,000 population) and kills about 200,000 people every year [6]. The incidence of typhoid in endemic areas is typically considered to be low in the first few years of life, peaking in school-aged children and young adults and then falling in middle age [7]. The high risk populations for *Salmonella* infections include young, elderly, pregnant woman, immune compromised and HIV infected individuals [8, 9]. Moreover, reports pertaining to typhoid fever during pregnancy are very few but have shown to cause serious maternal infection, with transplacental spread to the fetus [10]. Complications in pregnancy due to *Salmonella* infections include endomyometritis, salpingitis, chorioamnionitis, transplacental infection of the fetus, septic abortion, neonatal septicemia, and meningitis, and few reported cases of life-threatening septicemia in the mother [1]. Most notably, the host response to infections during pregnancy needs to be investigated so as to enable better management and to reduce the risk of transmission of the disease. Thus, the present study was designed to delineate the effect of typhoid in pregnant mouse model.

## Materials and Methods

### Bacterial strain and preparation of inoculum

*Salmonella enterica* serovar Typhimurium NCTC 74 (virulent strain) procured from Central Research Institute (CRI), Kasauli, India was maintained on nutrient agar slants by regular sub culturing at an interval of 15 days by incubating at 37 °C for 24 hours. The strain was examined biochemically and serologically prior to use. For preparation of inoculums, the culture was grown in nutrient broth for 9 hour at 37 °C. After the incubation, the cells were harvested by cold centrifugation at 2000 x g for 10 minutes, washed and suspended in phosphate buffer saline (PBS). Viable counts were determined by spread plating method on MacConkey agar followed by incubation at 37 °C and counting CFU 24 hours later.

### Animals

Crossbred female BALB/c mice, aged 5 - 6 weeks old, weighing 18 - 20 g were procured from the Central Animal House, Panjab University, Chandigarh, India. The mice were provided standard pellet diet (Hindustan Lever Ltd. Mumbai, India) and water *ad libitum*. These animals were monitored for any bacterial infection by streaking tail vein blood directly onto Mac Conkey agar/Nutrient agar. Care and use of animals were in accordance with the guidelines of the Panjab University animal ethical committee and experimental protocols were also approved by the animal ethics committee.

### Assessment of first gestational day

Female mice were mated with males of same strain in the ratio of 2:1 and were examined daily (morning and evening) for the presence of vaginal plug. The day vaginal plug observed was marked as the first gestational day (GD) [11].

### Experimental design and follow up of the experimental animals

Four groups were employed comprising of 6 - 7 animals in each group. Group I (Control) comprising of normal female animals. Group II (*Salmonella-*infected) female mice were challenged orally with 1 x 10^4^ cells of *S.* Typhimurium [12]. Group III (pregnant) comprising of pregnant mice which were fed orally with normal saline on GD 10. Group IV: (pregnant-*Salmonella-*infected) comprising of pregnant mice which were fed orally with 1 x 10^4^ cells of *S.* Typhimurium on GD 10. Mice belonging to all the groups were sacrificed on day 5 post infection and bacterial loads, liver enzymes (Aspartate aminotransferase (AST), Alanine aminotransferase (ALT), Alkaline phosphatase (ALP)), the levels of oxidant and antioxidant and pathological alterations in liver, spleen and placenta were studied.

### Preparation of tissue homogenates and post mitochondrial supernatant preparation

After sacrifying the animals, liver, spleen, and placenta were removed. Tissue homogenates were prepared in PBS using mechanically driven Teflon fitted Potter Elvejhem type homogenizer under ice cold condition for 30 - 45 seconds. Post mitochondrial supernatant was prepared by centrifugation of tissues homogenates at 400 x g for 10 minutes. The pellet was discarded and the supernatant was further centrifuged at 2,200 x g for 10 minutes at 4 °C. The supernatant was labeled as post mitochondrial supernatant preparation and used for the estimation of various oxidants and antioxidants level. Protein content in the tissue homogenates and PMS were estimated [13].

### Assesment of bacterial load

Bacterial load in the liver, spleen and placenta was assayed by plating 10 fold serial dilutions of tissue homogenates on MacConkey agar. The culture plates were incubated at 37 °C for 24 hours and colony forming units (CFU) were counted.

### Assay of liver damage markers

#### Estimation of serum aminotransferase (ALT and AST) and alkaline phosphatase (ALP)

Blood was collected by retro-orbital puncture from all the groups of mice before they were sacrificed for carrying out other estimations. ALT, AST and ALP from serum were analyzed by autoanalyser (Roche/Hitachi) and results were expressed as IU/L.

### Estimation of lipid peroxidation (malondialdehyde) levels

The amount of malondialdehyde (MDA), the end product of lipid peroxidation was measured in the liver, spleen and placenta as per the method of Wills [14]. In brief, 0.5 mL of Tris-HCl buffer (0.1 M, pH 7.4) was added to 0.5 mL of tissue homogenates and kept at 37 °C for 2 hours. Following incubation, 1.0 mL of 10% (w/v) trichloroacetic acid (ice-cold) was added and the mixture was centrifuged at 200 x g for 10 minutes. To 1.0 mL of supernatant, 1.0 mL of 0.67 % (w/v) thiobarbituric acid was added and kept in boiling water bath for 10 minutes. After cooling the tubes, 1.0 mL of distilled water was added and absorbance was measured at 532 nm. The results were expressed as micromoles of MDA per milligram of protein, using the molar extinction coefficient of chromophore (1.56 x 10^5^ M^-1^ cm^-1^).

### Estimation of reduced glutathione (GSH)

GSH levels in various tissues were estimated as per Ellman [15]. One milliliter of tissue homogenate was precipitated with 1.0 mL of 4% sulphosalicyclic acid. The sample was kept at 4 °C for at least 1 hour, and was centrifuged at 100 x *g* for 15 minutes at 4 °C. The assay mixture contained 0.1 mL of supernatant, 0.2 mL of 0.01 M dithionitro benzoic acid (DTNB) and 2.7 mL of phosphate buffer (0.1 M, pH 8.0) in total volume of 3.0 mL. The mixture was kept at room temperature for 10 minutes and was measured at 412 nm. The results were expressed as micromoles of GSH/milligram of protein.

### Estimation of superoxide dismutase (SOD) activity

SOD activity was assayed according to the method of Kono [16]. Briefly, the reaction was initiated by addition of 0.5 mL of hydroxylamine hydrochloride to the reaction mixture containing 2.0 mL nitroblue tetrazolium (NBT) and 0.1 mL PMS of tissue homogenate. SOD activity was expressed as units of SOD per milligram of protein where one unit activity is defined as the amount of SOD required to inhibit the rate of reduction of NBT by 50%.

### Estimation of catalase activity

Catalase activity was assayed by the method of Claiborne [17]. The assay mixture consisted of 1.95 mL phosphate buffer (0.05 M, pH 7.0) 1.0 mL of hydrogen peroxide and 0.05 mL of PMS in a final volume of 3.0 mL. Change in absorbance was recorded spectrophotometrically at 240 nm. The results were expressed in terms of K min^-1^, where ‘K’ is the amount of enzyme which liberates half the peroxide oxygen from H_2_O_2_ of any concentration in 100 seconds at 25 °C.

### Histopathological studies

Mice were sacrificed by retro orbital plexus bleeding and organs were removed aseptically, fixed in 10% buffered formalin. Tissues were dehydrated in different grades of alcohol i.e. 70%, 80%, 90% and absolute alcohol for 30 minutes, 40 minutes and 1 hour respectively, followed by washing in xylene for 1 hour each at room temperature. Finally, the tissues were dipped in molten paraffin wax and were quickly cooled to prevent crystallization. Thin sections of tissue were cut and embedded tissue sections were kept in a water bath at 50 °C to remove the wax. Sections were mounted on separate clean glass microscope slides and stained with haematoxylin and eosin stain (H and E stain). The slides were blot-dried, mounted with Distyrene Plasticizer Xylene (DPX) and were examined by light microscopy.

### Statistical analysis

Results were expressed as mean ± standard error. The inter group variation was assessed by one way analysis of variance (ANOVA) followed by Dunnett’s multiple comparison test. Statistical significance of the results was calculated at P < 0.05.

## Results

### Bacterial load

**Figure 1:**
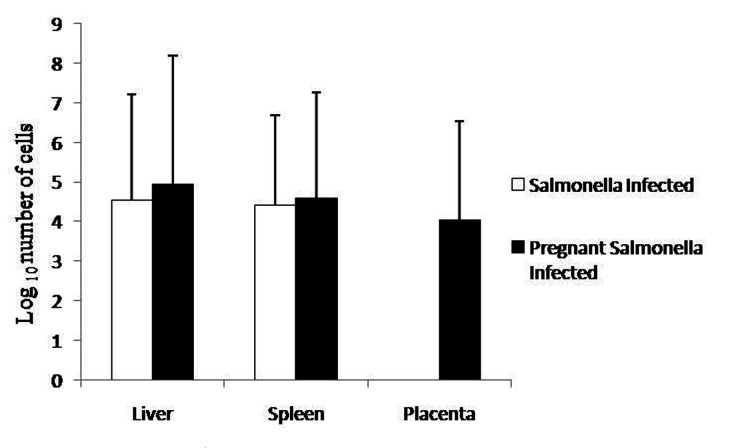
Bacterial Load in different organs. Values are expressed in log_10_ number of cells as Mean ± S.E.

Pregnant mice infected with *Salmonella* Typhimurium on GD-10 had slightly higher bacterial load in the liver and spleen compared with *Salmonella-*infected mice as observed by plating tissue homogenates on MacConkey agar. However, no significant difference was observed in the bacterial load in placenta compared with liver and spleen of pregnant-*Salmonella*-infected mice (Fig. 1).

### Levels of liver damage markers

**Figure 2:**
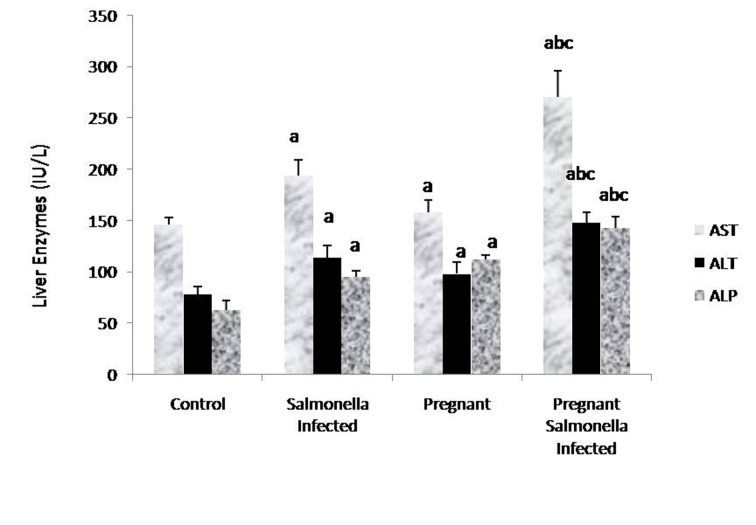
Liver enzymes (IU/L) in various groups. Values are expressed as Mean ± S.E. ‘a’ shows significant difference (P < 0.05) from control mice; ‘b’ shows significant difference (P < 0.05) from pregnant mice; ‘c’ shows significant difference (P < 0.05) from Salmonella-infected mice.

The levels of AST, ALT and ALP in the serum samples of mice belonging to *Salmonella*-infected and pregnant-*Salmonella*-infected were significantly (P < 0.05) higher compared with counter control groups. Interestingly, AST, ALT and ALP levels were significantly increased (P < 0.05) in pregnant *Salmonella*-infected mice compared with *Salmonella*-infected mice (Fig. 2).

### Estimation of lipid peroxidation (MDA) levels

**Figure 3:**
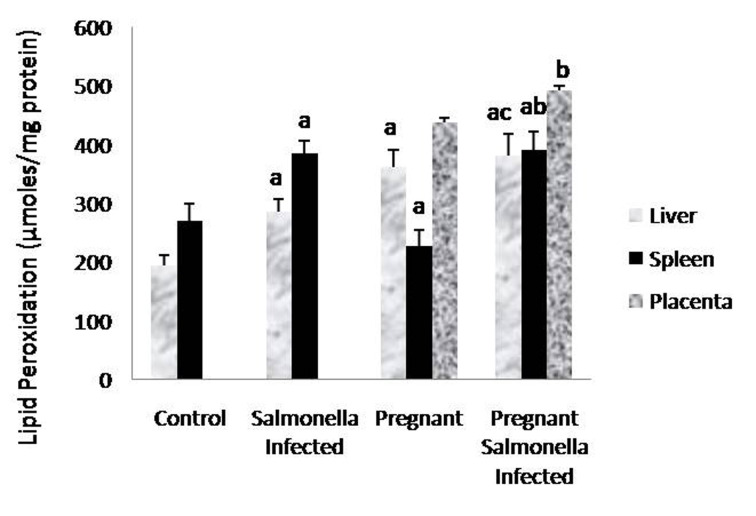
Lipid peroxidation (µmol/mg protein) in liver, spleen and placental homogenates of mice belonging to different groups. Values are expressed as Mean ± S.E. ‘a’ shows significant difference (P < 0.05) from control mice; ‘b’ shows significant difference (P < 0.05) from pregnant mice; ‘c’ shows significant difference (P < 0.05) from Salmonella-infected mice.

The levels of malondialdehyde were significantly (P < 0.05) higher in *Salmonella*-infected and pregnant-*Salmonella*-infected mice compared with counter control groups. Interestingly, the levels of MDA in placenta were also higher in pregnant-*Salmonella*-infected mice compared with pregnant mice (Fig. 3).

### Levels of antioxidants

**Figure 4:**
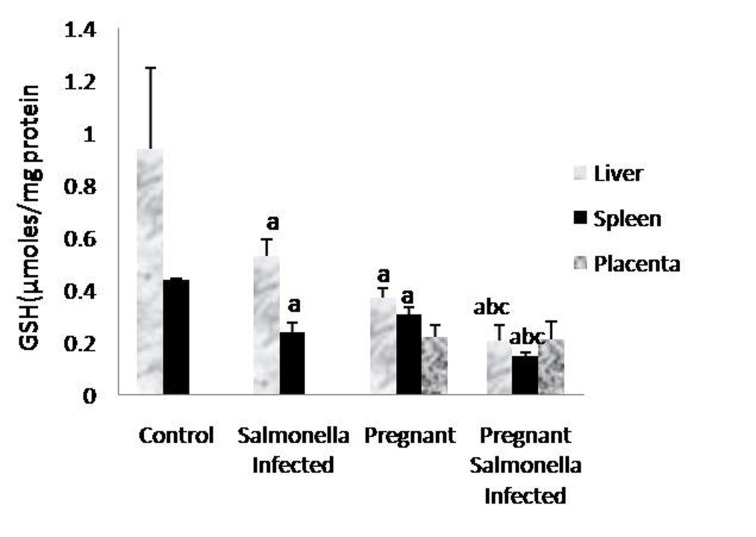
GSH levels (µmol/mg protein) in liver, spleen and placental homogenates of mice belonging to different groups. Values are expressed as Mean ± S.E. ‘a’ shows significant difference (P < 0.05) from control mice; ‘b’ shows significant difference (P < 0.05) from pregnant Salmonella-infected mice; ‘c’ shows significant difference (P < 0.05) from Salmonella-infected mice.

**Figure 5:**
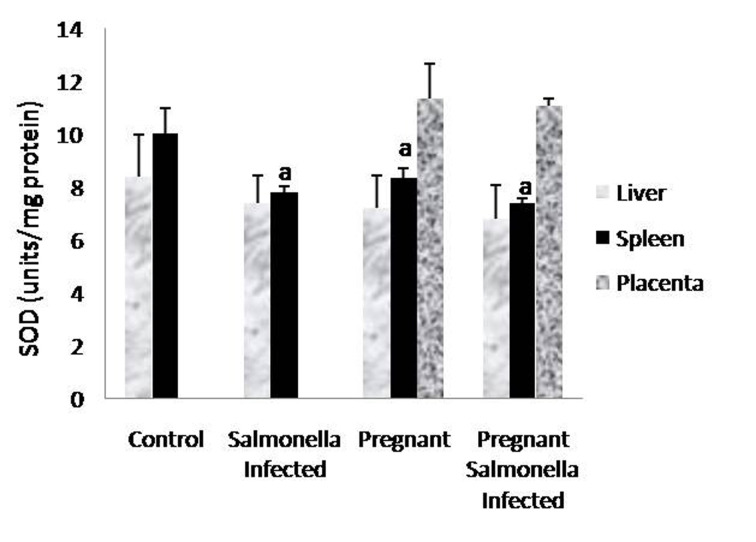
Activity of superoxide dismutase (units/mg protein) in liver, spleen and placental homogenates of mice belonging to different groups. Values are expressed as Mean ± S.E. ‘a’ shows significant difference (P < 0.05) from control mice.

**Figure 6:**
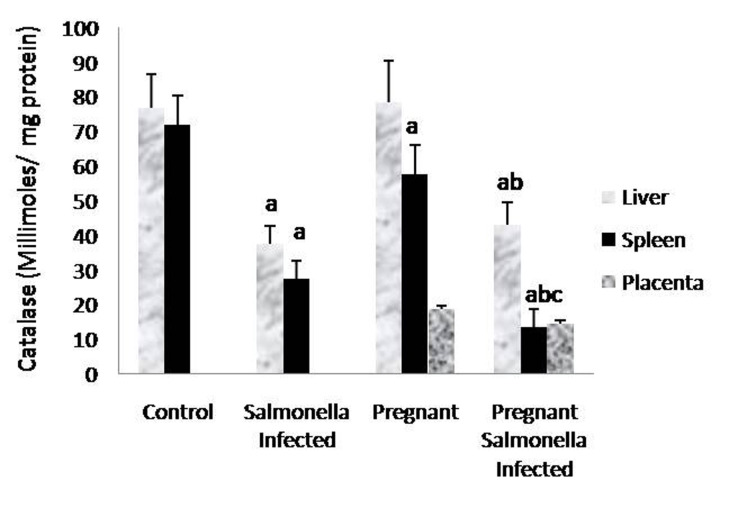
Catalase activity (Kmin^-1^) in liver, spleen and placental homogenates of mice belonging to different groups. Values are expressed as Mean ± S.E. ‘a’ shows significant difference (P < 0.05) from control; ‘b’ shows significant difference (P < 0.05) from pregnant mice; ‘c’ shows significant difference (P < 0.05) from Salmonella-infected mice.

The GSH levels in liver, spleen and placenta were significantly decreased (P < 0.05) in *Salmonella-*infected, pregnant and pregnant-*Salmonella*-infected mice compared with control mice, but the decrease was maximum in pregnant *Salmonella*-infected mice (Fig. 4). However, no significant difference was observed in SOD activity in the liver and placenta of mice belonging to all groups except the spleen where the activity of SOD was decreased significantly (P < 0.05) compared with control mice (Fig. 5). The catalase activity was found to be significantly decreased (P < 0.05) in the liver and spleen of *Salmonella*-infected and pregnant-*Salmonella*-infected mice compared with their counter controls (Fig. 6).

### Histopathological studies

**Figure 7:**
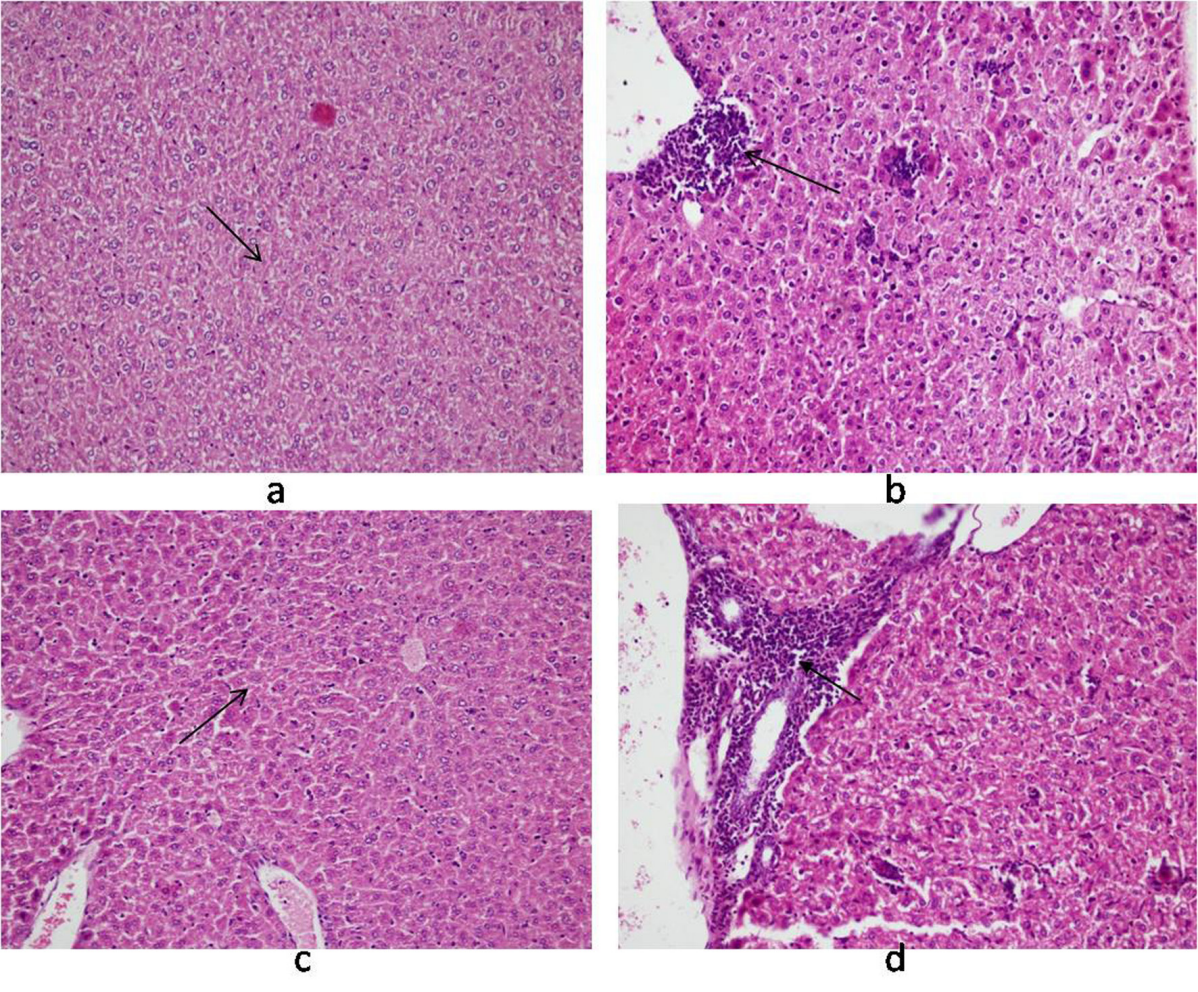
Photomicrograph of liver: (a) control mice (Group I) showing normal hepatocytes; (b) Salmonella-infected mice (Group II) showing lobular and inflammation of cells (arrow); (c) pregnant mice (Group III) showing Kupffer cells; (d) pregnant-Salmonella-infected mice (Group IV) showing portal tract inflammation (arrow). H and E stain, 100 x.

**Figure 8:**
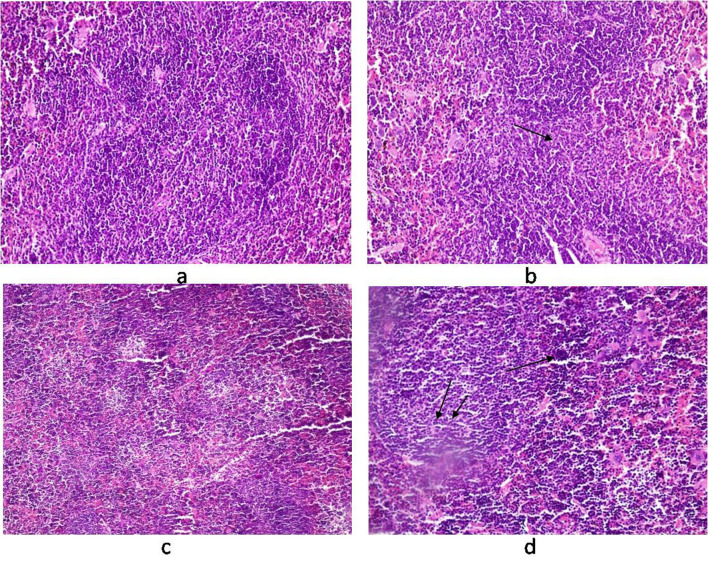
Photomicrograph of spleen. (a) control mice (Group I) showing normal texture of splenocytes; (b) Salmonella-infected mice (Group II) showing expansion of lymphoid tissue (arrow); (c) pregnant mice (Group III) showing normal structure of white cells with some expansion of red pulp; (d) pregnant-Salmonella-infected mice (Group IV) showing large lymphoid tissue (double arrow) and extramedullarly hematopoisis (single arrow) with mild excess of megakaryocytes. H and E stain, 50 x.

**Figure 9:**
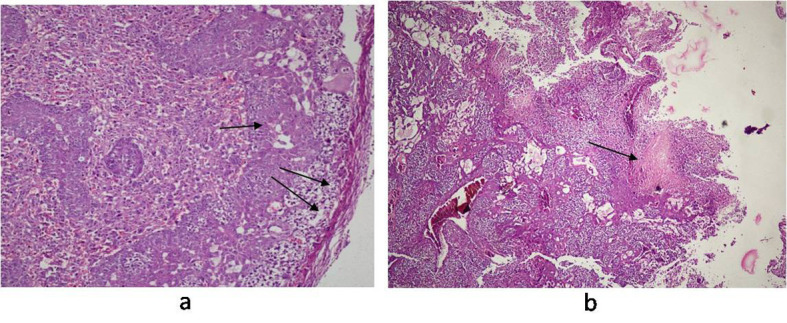
Photomicrograph of Placenta: (a) pregnant mice (Group III) showing normal structure of trophoblastic membrane (arrow) and syncytiotrophoblastic membrane (double arrow); (b) pregnant-Salmonella- infected mice (Group IV) showing altered structure and large foci of necrosis (arrow). H and E stain, 50 x.

Histology of the liver sections from *Salmonella-*infected mice showed lobulated inflammed cells, damaged central portal vein, hepatocytic destruction in comparison with normal hepatocytes in control mice (Fig. 7a, b) and mild increased Kupffer cells in pregnant mice (Fig. 7c). In contrast, the liver of pregnant-*Salmonella*-infected mice had inflammed portal tract and enlarged nucleus (Fig. 7d). The spleen of both control and pregnant mice had normal splenocytes but pregnant mice had enlarged red pulp area (Fig. 8a, c), while, *Salmonella*-infected mice had expanded lymphoid tissue (Fig. 8b) compared with large lymphoid tissue and extramedullarly hematopoiesis along with mild excess of megakaryocytes in pregnant-*Salmonella-*infected mice (Fig. 8d). The placenta of pregnant-*Salmonella-*infected mice has large foci of necrosis surrounded by karyorrhectic debris and neutrophil infiltration compared with normal trophoblastic and syncytiotrophoblastic membrane in pregnant mice (Fig. 9a, b).

## Discussion

Typhoid fever is still a disease of major importance, caused by *Salmonella* contaminated food and drink, spares no age or sex and poses high risks to the pregnant women. Since, very few reports are available pertaining to typhoid fever in pregnancy and its effect on physiology of pivotal organs, the present study was designed to assess the effect of *Salmonella* infection in pregnant mice with respect to bacterial load, oxidant and anti-oxidant levels vis-a-vis histological alterations.

The exacerbated bacterial load in the liver, spleen and placenta of pregnant-*Salmonella-*infected mice could be due to hormonal changes and reduced systemic innate immunity and is in accordance with earlier observation [1, 12, 18]. It can be said that though the placenta is permissive to *S*. Typhimurium proliferation but the reduced number may probably be due to environmental differences from the liver and spleen that affects the doubling time. As Chatopadhaya et al have shown that *S.* Typhimurium exhibits contrasting intracellular replication rates depending the cell type, doubling in about 1 hour in choriocarcinoma cells and 4 hours in Hela cells in vitro [3].

The nature of physiological response evoked by the pathogens during pregnancy appears to be the key mechanism that interfere the pathogenesis of infection and the host pathogen relationship. We have found that the alanine, aspartate aminotransferases and alkaline phosphatase, the biochemical markers for liver damage were higher in both the infected groups of mice and may be attributed to the liver damage, occurring either due to hepatic granulomas or hepatic abscesses that may release these liver enzymes into serum and corroborate with earlier studies [19, 20]. The enhanced levels of ALP in pregnant mice are due to placenta, a metabolically active tissue and rich source of ALP [21].

Oxidative stress is mediated by reactive oxygen species (ROS) and is the important mediators of tissue injury in various diseases e.g. typhoid, anemia in malaria and CNS disorder [18, 22, 23]. The enhanced levels of MDA in *Salmonella*-infected, pregnant and pregnant-*Salmonella*-infected mice are responsible for the observed damage in the liver, spleen and placenta and are in accordance with earlier findings [12, 18]. Moreover, infections and associated responses are considered to play a pivotal role in placental pathology and increased oxidative stress in pregnant-*Salmonella*-infected mice may be due to the mitochondrial rich placenta [24].

The development of tissue injury and outcome of the disease depends upon the balance between the generation of toxic radicals and tissue antioxidant status [18, 25]. GSH, an important cellular antioxidant acts either by protecting cells from lipid peroxidation or by protecting protein sulfhydryl group from being oxidized by these radicals [26, 27]. A significant decrease in GSH levels was observed in all the organs of test groups and is in agreement with earlier findings [28, 29]. Furthermore, the observed decreased antioxidant enzymes (SOD and catalase) activity may be due to hampered dismutation of superoxide anions and inefficient detoxification of H_2_O_2_ resulting in the formation of hydroxyl radicals that in turn enhanced the lipid peroxidation resulting into anatomical alterations in the liver, spleen and placenta of pregnant mice. The loss of structural integrity produced by *S.* Thyphimurium correlated with the wide spread neutrophil infiltration as neutrophils are considered vital for defense against *S*. Typhimurium but over activation resulted into deleterious effects in host [3].

### Conclusion

The present study clearly indicates that infection with *S.* Typhimurium during pregnancy leads to severe tissue damage, due to increased bacterial burden and liver enzymes leading to profound levels of lipid peroxidation and decreased activities of antioxidants. Thus, can be concluded that the mouse model of *S.* Typhimurium infection may mimics human typhoid, causing disseminated disease. Also typhoid fever during pregnancy is more severe due to profound intracellular proliferating ability of *Salmonella* species in various pivotal organs that triggers the oxidative response and reduced antioxidants leading to organs necrosis that may adversely influence the materno-fetal outcome.
